# Microbial Interventions to Control and Reduce Blood Pressure in Australia (MICRoBIA): rationale and design of a double-blinded randomised cross-over placebo controlled trial

**DOI:** 10.1186/s13063-021-05468-2

**Published:** 2021-07-27

**Authors:** Dakota Rhys-Jones, Rachel E. Climie, Paul A. Gill, Hamdi A. Jama, Geoffrey A. Head, Peter R. Gibson, David M. Kaye, Jane G. Muir, Francine Z. Marques

**Affiliations:** 1 Hypertension Research Laboratory, School of Biological Sciences, Faculty of Science, Monash University, 25 Rainforest Walk, Clayton, Victoria 3800 Australia; 2 Department of Gastroenterology, Central Clinical School, Monash University, Melbourne, Australia; 3 Sports Cardiology, Baker Heart and Diabetes Institute, Melbourne, Australia; 4 Menzies Institute for Medical Research, University of Tasmanian, Hobart, Australia; 5 Neuropharmacology Laboratory, Baker Heart and Diabetes Institute, Melbourne, Australia; 6 Department of Pharmacology, Monash University, Melbourne, Australia; 7 Heart Failure Research Group, Baker Heart and Diabetes Institute, Melbourne, Australia; 8 Central Clinical School, Faculty of Medicine Nursing and Health Sciences, Monash University, Melbourne, Australia; 9 Department of Cardiology, Alfred Hospital, Melbourne, Australia

**Keywords:** Hypertension, Blood pressure, Fibre, Diet, Prebiotics, Postbiotics

## Abstract

**Background:**

Hypertension is a prevalent chronic disease worldwide that remains poorly controlled. Recent studies support the concept that the gut microbiota is involved in the development of hypertension and that dietary fibre intake may act through the gut microbiota to lower blood pressure (BP). Resistant starch is a type of prebiotic fibre which is metabolised by commensal bacteria in the colon to produce short-chain fatty acids (SCFAs), including acetate, propionate, and butyrate. Previous work in pre-clinical models provides strong evidence that both prebiotic fibre as well as SCFAs (i.e. postbiotics) can prevent the development of hypertension. The aim of this clinical trial is to determine if acetylated and butyrylated modified resistant starch can decrease BP of hypertensive individuals via the modulation of the gut microbiota and release of high levels of SCFAs.

**Methods:**

This is a phase IIa double-blinded, randomised, cross-over, placebo controlled trial. Participants are randomly allocated to receive either a diet containing 40 g/day of the modified resistant starch or placebo (corn starch or regular flour) for 3 weeks on each diet, with a 3-week washout period between the two diets. BP is measured in the office, at home, and using a 24-h ambulatory device. Arterial stiffness is measured using carotid-to-femoral pulse wave velocity. Our primary endpoint is a reduction in ambulatory daytime systolic BP. Secondary endpoints include changes to circulating cytokines, immune markers, and modulation to the gut microbiome.

**Discussion:**

The findings of this study will provide the first evidence for the use of a combination of pre- and postbiotics to lower BP in humans. The results are expected at the end of 2021.

**Trial registration:**

Australia and New Zealand Clinical Trial Registry ACTRN12619000916145. Registered on 1 July 2019.

## Background

High blood pressure (BP), or hypertension, is a highly prevalent chronic disease, affecting one in three people worldwide [1]. Hypertension remains poorly controlled due to low compliance and medication adherence [2, 3]. The pathogenesis of hypertension and BP control is complex: combined with genetic and environmental factors, recent studies now support the concept that the gut microbiota is involved in the development and maintenance of high BP [4]. Epidemiological studies suggest that dietary components such as fibre may mitigate the development of hypertension [5], and interventions to increase fermentable and non-fermentable fibre intake significantly reduced systolic and diastolic BP in patients with hypertension [4, 6, 7].

Results from pre-clinical studies support resistant starch is a type of dietary fibre that has potential to exert different beneficial outcomes for cardiovascular disease (CVD) [8], yet has been neglected in clinical trials. These starches are considered prebiotic (i.e. substrates for stimulating the growth of certain beneficial gut bacteria), as they resist digestion in the upper gastrointestinal tract and pass undigested to the large intestine, where they are fermented by commensal bacteria. Diets rich in prebiotic fibre increase gut microbiota populations that generate short-chain fatty acids (SCFAs), such as acetate, propionate, and butyrate.

Earlier studies have found resistant starch intakes in both Australia and the USA are low (approximately 3–9 g per person per day) [9, 10]. Resistant starches are heterogeneous in nature, and there are no current dietary recommendations regarding intake. However, some have suggested intake of 6 g per meal, or 20 g per day, will promote health benefits, such modulation of post-prandial glucose and insulin levels [9]. High amylose maize starches (HAMS) are made up of approximately 50% resistant starch and are used in food products in Australia (e.g. breads, cereals) and can also be purchased as a standalone supplement [11]. Thus, there is a case for a diet high in HAMS and, therefore, resistant starch or prebiotic fibre to exert positive health effects to individuals and of interest, those with hypertension.

The relationship between diet, gut microbiome modulation, and the availability of SCFAs is believed to be the key to the ability of a high prebiotic fibre diet to ameliorate inflammatory diseases [12–14]. Early ex vivo studies showed that acetate and butyrate mediate concentration-dependent dilatation of ventral tail artery in rats and human colonic resistance arteries [15, 16]. These are consistent with findings by us and others supporting that a diet high in resistant starches [8] or direct supplementation with acetate [8], propionate [17], and butyrate [18] prevent the development of high BP and its cardio-renal complications in pre-clinical models. In pre-clinical models, lack of resistant starches lead to the development of a hypertensinogenic gut microbiota in germ-free mice [19]. Combined, these studies suggest that the use of prebiotic resistant starches and postbiotics (i.e. SCFAs) may lower BP, but translational evidence in humans are lacking. Here, we describe our ongoing world-firstproof-of-concept phase IIa clinical trial, the Microbial Interventions to Control and Reduce Blood Pressure in Australia (MICRoBIA), where we are assessing the use of an acetylated and butyrylated high amylose maize starch (HAMSAB) as a treatment for hypertension.

## Methods

### Aims and hypotheses

It is hypothesised that the gut microbiota and their metabolites acetate and butyrate prevent the development of hypertension and that medicinal food containing prebiotic fibres and postbiotics (i.e. acetate and butyrate), such as HAMSAB, can be used to lower BP. Thus, our aim is to determine if HAMSAB, which produces high levels of SCFAs as well as leading to the release of high levels of acetate and butyrate as a result of microbial fermentation could be used as a new strategy to lower BP.

### Study design

**Fig. 1:**
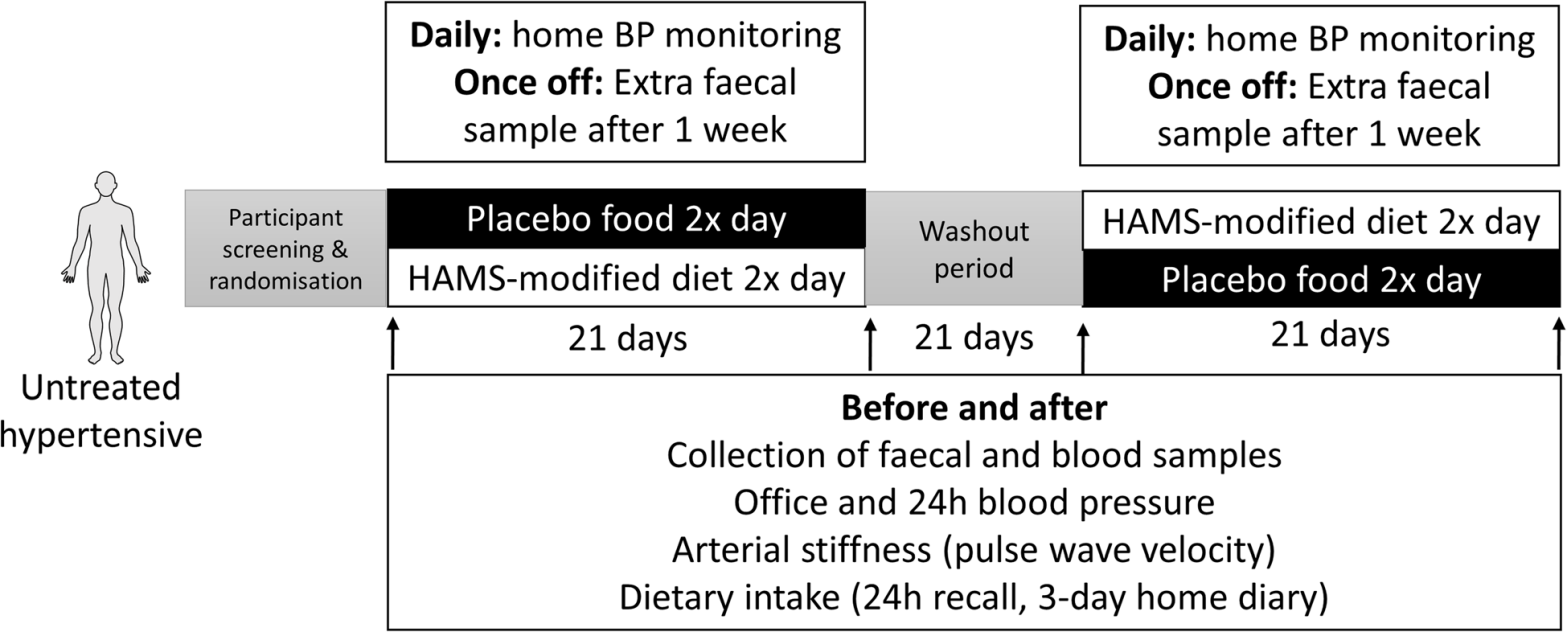
Design of our double-blind, cross-over, randomised trial. BP, blood pressure

This is a double-blinded, randomised, cross-over, placebo controlled trial as shown in Fig. 1.

### Recruitment summary

Recruitment is being conducted through advertisement placed around the Alfred Hospital (Melbourne, Australia) precinct as well as public places (GP offices, shopping malls/centres, noticeboards) in this locality. Previous research participants who approved to be contacted for future research were also contacted via email through a large database to be considered for the trial. Online social media platforms are also used to recruit participants (Monash FODMAP platforms, Marques Lab, Facebook, Twitter, Google ads). The trial gained exposure through an article in a tabloid based in Melbourne (The Herald Sun) as well as an article through a Monash University online news platform (Monash Lens). Recruitment started in July 2019 and is estimated to finish in October 2021.

### Study population

**Fig. 2:**
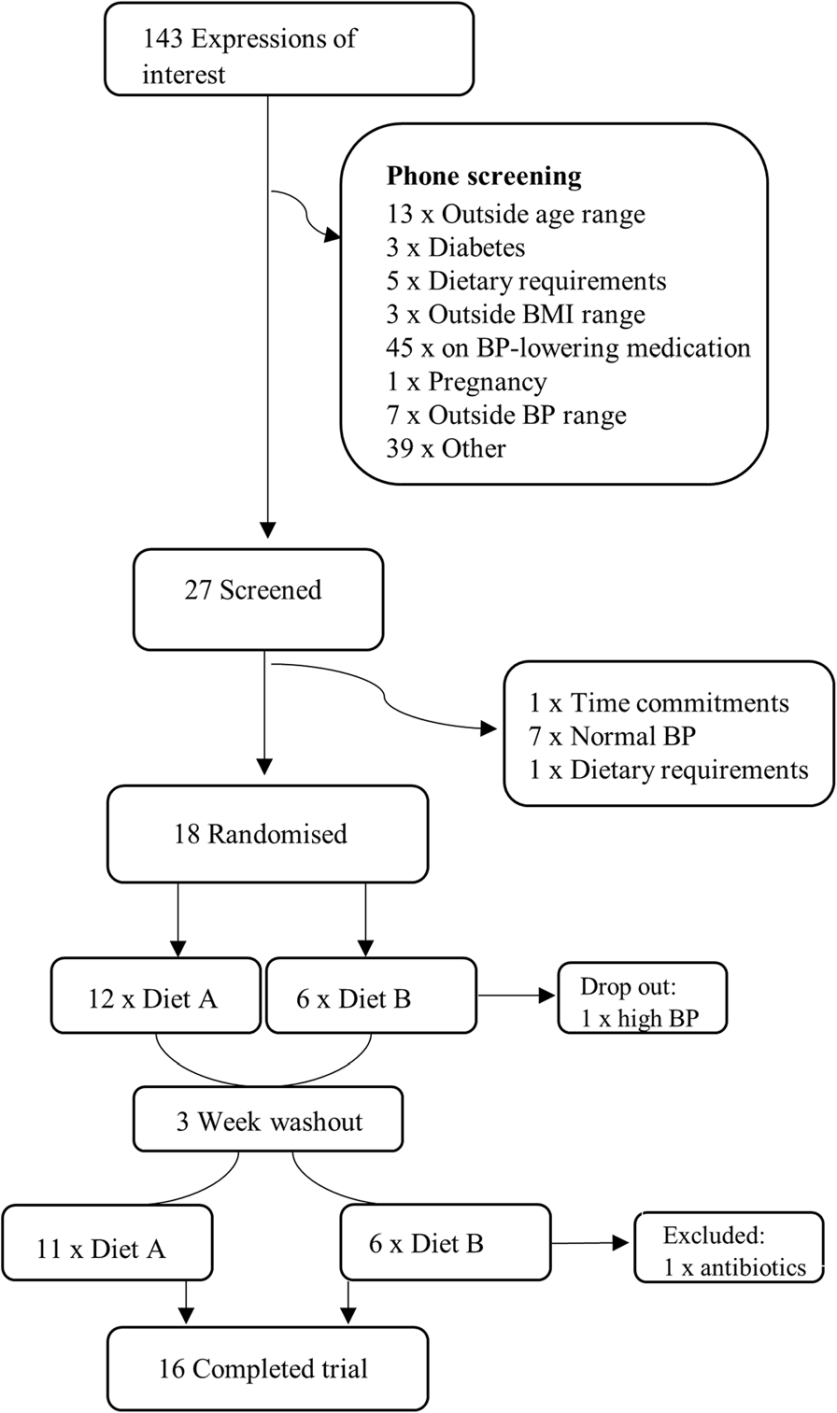
Recruitment summary. Out of 143 expressions of interest, 16 participants have finished the trial so far. BMI, body mass index; BP, blood pressure

Males and females with untreated hypertension as defined by the Australian National Heart Foundation guidelines [office BP ≥ 140/90 mmHg and 24 h ambulatory blood pressure monitoring (ABPM) ≥ 130/80)] are being recruited for this study. Participants with masked hypertension are included and defined as office BP ≤ 140/90 mmHg but ABPM ≥ 130/80. Participants are also required to have a BMI of 18.5–35 kg/m^2^, due to the association between weight and the gut microbiota [20]. Exclusion criteria include the use of anti-hypertensive medication, office BP ≥ 165/100 mmHg, recent use of antibiotics (< 3 months) or probiotics (< 6 weeks), type 1 or 2 diabetes, pregnancy, and the presence of gastrointestinal diseases. Participants are also excluded if there are any dietary requirements (e.g. vegetarian and coeliac). Figure 2 describes the current study population at time of publication.

### Sample size

In this proof-of-principle study, we estimate we will require 33 subjects to achieve 80% power with *α* = 0.05 (calculated effect size 0.5) to determine a 5 mmHg difference in ambulatory daytime systolic BP after intake of modified HAMS for 3 weeks. The 3 weeks intervention was chosen based on previously published papers [8, 19]. We will aim to recruit 38 participants per group to allow for a 20% drop out rate. We have recruited 17 participants to date, with one dropout due to BP > 165/100 and requiring medication. One participant had to be excluded after completion of the trial due to antibiotic intake needed whilst in the second arm of the study, resulting in 16 participants that have completed the trial. Due to the SARS-COV-2 pandemic, we were forced to stop the trial several times, which has caused several delays in recruitment.

### Trial status

We are currently recruiting participants.

### Study protocol and measurements

A diagram of the study protocol (version 2, date 27 May 2019) is presented in Fig. 1. Participants are randomly stratified based on age, sex and BMI using REDCap (Version 9.1.0 Tennessee) to either a diet containing 40 g HAMSAB or placebo maize-starch daily by the study coordinator. REDCap is also used to manage all the information by the study coordinator. The choice of use of a placebo food instead of BP-lowering drug intervention addresses the issue that participants who volunteered to our study did not want to take medication in the first place. Both the study participants and study coordinator are blinded to the diet containing the prebiotic supplement (thus, they are referred to Diet A and Diet B in the Figures). The study period is 9 weeks: 3 weeks actively on either diet, with a 3-week washout period in between. The purpose of the washout period is to allow the gut microbiota and levels of acetate and butyrate to return to baseline. These will be confirmed in samples collected in the third visit compared to the first visit. The washout period was determined based on previous gut microbiota studies with a similar design [21], based on the fact that the gut microbiota can change rapidly (< 7 days) [22]. Since this is a cross-over study, data will be compared between visits one and two, and visits three and four, minimising the effect if we find that the effect of HAMSAB lasted longer than 3 weeks in those randomised into the intervention arm first. Participants are provided 2 meals daily and are instructed to eat their usual foods around the trial’s food. The dietary intake of the participants is not restricted, and the foods provided to them are suggested to in place of normal foods (i.e. replacing a usual breakfast of toast with a ‘breakfast muffin’). To increase compliance, we have opted to not replace every meal. A sample 7-day meal plan that is cycled during the 3-week diet phase is shown in Table 1. Participants are provided with a list of foods containing natural resistant starches and SCFAs to avoid during the study period (e.g. green banana flour, waxy maize starch, kombucha, vinegars). Participants are provided with a food diary for the duration of the study, where they document approximate intake in quartiles (i.e. 0, ¼, ½, ¾, all). This data is then translated for nutritional analysis using FoodWorks Professional Software (Version 7.01, Xyris, Queensland).

**Table 1:** Seven-day meal plan supplied for participants for 3 weeks

	Day 1	Day 2	Day 3	Day 4	Day 5	Day 6	Day 7
**AM meal**	Raspberry and white chocolate muffin	Breakfast muffin	Lemon and poppyseed muffin	Savoury muffin	Raspberry and white chocolate muffin	Breakfast muffin	Savoury muffin
**PM meal**	Arancini balls	Tuna burger	Frittata	Beef burger	Arancini balls	Tuna burger	Beef burger

Participants are initially screened over the phone to assess eligibility according to the inclusion and exclusion criteria. Those that meet the inclusion criteria are invited to attend a baseline assessment where signed consent is obtained. At the baseline assessment, participants spend approximately 60–90 min with the researcher for all BP measurements, including testing of office BP, carotid-to-femoral pulse wave velocity, as well as providing the 24 h ABPM device and home monitoring device. The researcher discusses the 3-day food diary with the participants, collects the 24-h dietary recall, and provides the faecal tubes with instructions for collection.

Primary outcome is a decrease in systolic BP. Office BP is measured under resting conditions (> 5 min sitting), with the researcher not in the room and an average of three measurements is taken using an automated digital BP monitor (Omron Healthcare, Japan, HEM-907). During the office baseline BP measurements, participants are seated with their back supported, arms and legs uncrossed, and not speaking. Participants are also instructed on how to correctly measure their BP at home according to the Australian Heart Foundation guidelines, and they are provided with a calibrated and research approved BP monitor (Omron Healthcare, Japan, HEM-7121) for the duration of the study for home BP measurements. At home, participants take two readings at the same time each day under rested conditions whilst on either diet which they document in a BP diary. Participants are also instructed to document any factors that they believe may affect their BP in this diary (e.g. medication changes, illnesses, severe stress).

Secondary outcome is a decrease in arterial stiffness. Measurements of carotid-femoral pulse wave velocity and pulse wave analysis are performed in duplicate using the Sphygmocor XCEL device (AtCor Medical, Sydney, NSW). Participants are seated and rested for the brachial measurements of pulse wave analysis and in the supine position for the pulse wave velocity measurement. Mobil-O-Graph (I.E.M Industrielle Entwicklung Medizintechnik GmbH) BP monitoring devices are used for the 24 h ABPM measurements. This takes BP measurements every 15 min during the day time and 30 min during the night time period. Night and day time periods are confirmed by a diary completed by the subject. Once the ABPM monitor has been returned to the researcher and hypertension has been confirmed, participants are provided with study food. Participants receive letters with their BP results which suggest when they should follow-up with their medical team, according to the National Heart Foundation of Australia BP guidelines.

Three-day food diaries and 24-h food recalls are used to understand habitual dietary intake amongst the study participants at each visit. Three-day food diaries are to be completed over two week days and one weekend day. This dietary information is recorded in FoodWorks Professional Software (Version 7.01, Xyris, Queensland) to calculate the average energy and macronutrient intake for each participant. Quantification of resistant starch is calculated from the Monash University FODMAP database and resistant starch report [23]. Participants are also instructed to follow the Australian Government’s ‘Eat for Health’ guidelines on alcohol, which limits alcohol to no more than two standard drinks on any day, and no more than four standard drinks on any occasion. Participants also complete the Visual Analogue Scale (VAS) questionnaire for gastrointestinal symptoms (e.g. abdominal symptoms, pain, wind, nausea), and a daily bowel symptom diary.

BMI and waist-to-hip ratio are measured at each visit. Patients are instructed to not plan any intended weight loss or weight gain strategies during the duration of the study, due to the effect of weight changes on BP. Participants also record the amount and types of physical activity they have participated in, during the week prior to each visit.

A fasting blood sample is taken on each visit and all testing is performed at the site hospital (The Alfred Hospital, Melbourne). Standard biochemical testing is performed including fasting blood glucose and fasting lipids and blood electrolytes and liver enzyme tests to determine renal and liver function (including eGFR, creatinine, total protein, albumin, globulin, bilirubin, ALT, GGT, ALP).

**Fig. 3:**
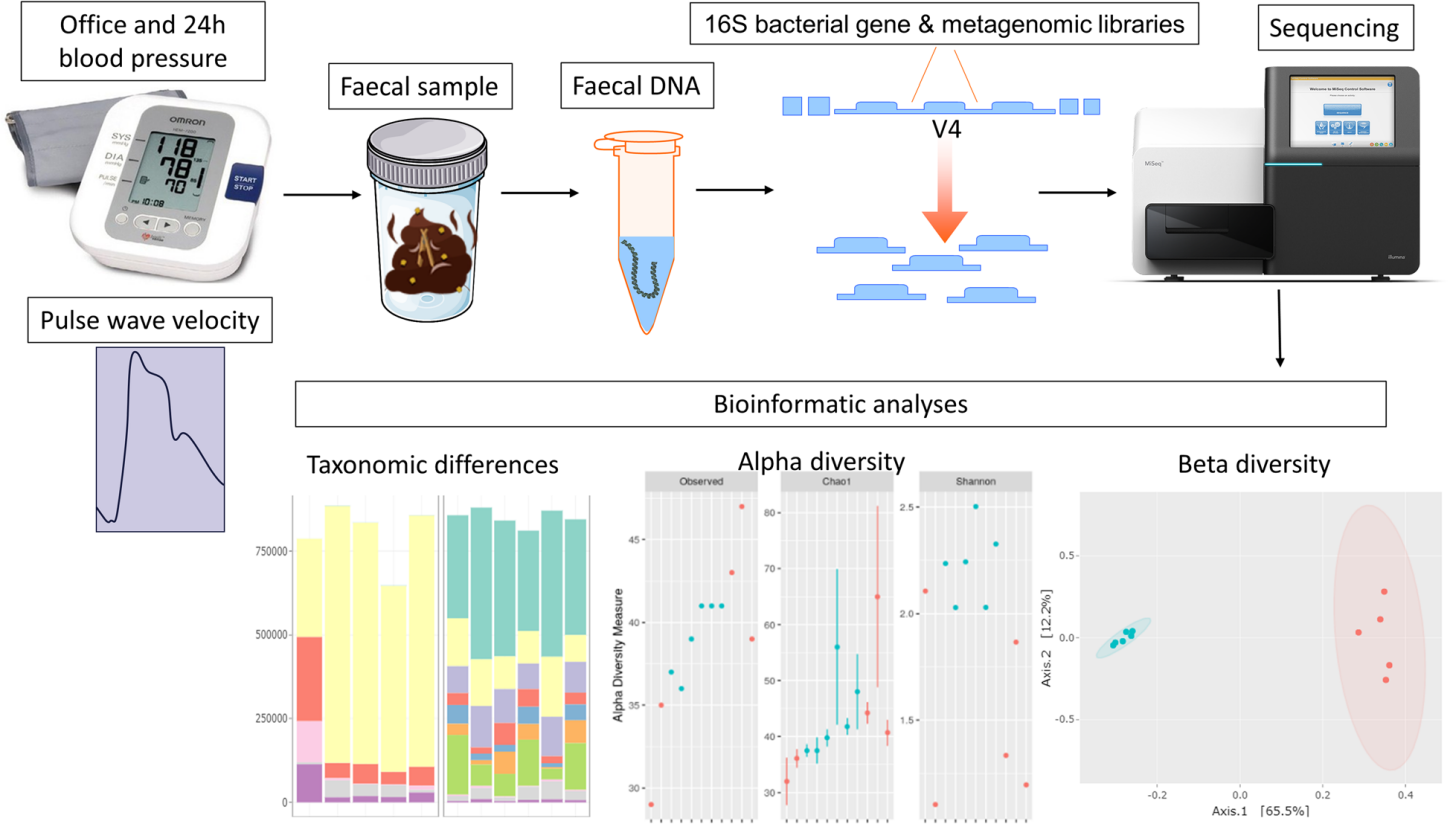
Collection and analyses of the gut microbiome. Following measurement of blood pressure (office and 24 h ambulatory blood pressure monitoring) and pulse wave velocity, participants collect a faecal sample. The DNA is then extracted; library targeting the 16S bacterial gene or whole-metagenome is prepared and sequenced. Bioinformatics analyses are used to determine taxonomic differences (before/after placebo and intervention), alpha diversity (i.e. within sample diversity), and beta diversity (i.e. between sample diversity). Plots are only representative

The third outcome is a change in levels of SCFAs. Plasma and faecal SCFAs will be measured at the end of the study using gas chromatography in triplicates, as previously described [24]. Faecal DNA preserved in DNA/RNA Shield and stored at – 80 °C will be extracted using the QIAamp PowerFecal DNA kit (Qiagen). The V4 region of the bacterial 16S rRNA will be amplified by PCR using 20 ng of DNA, Platinum Hot Start PCR master mix (ThermoFisher Scientific), 515F and 926R primers (Bioneer), and methods previously described [25] in a Veriti Thermal Cycler (ThermoFisher Scientific). Two hundred and forty nanograms of the product will be pooled, cleaned, and then sequenced in an Illumina MiSeq sequencer (300 bp paired-end reads, with minimum 100,000 reads per sample). 16S data will be analysed using the QIIME2 workflow [26]. We will analyse faecal samples collected from before, during (7 days) and after the interventions in terms of alpha diversity (i.e. number of bacteria and distribution within samples), beta diversity (i.e. types of bacteria and prevalence between samples), and taxonomic changes according to recent guidelines [27] (Fig. 3). This will inform further metagenomics analyses.

The study coordinator and the principal investigators meet weekly or fortnightly to monitor progress. Our studied followed the SPIRIT reporting guidelines [28].

### Adverse effects

Adverse effects would be reported to the Monash University Human Research Ethics Committee. Due to the use of diet as our intervention, adverse events are uncommon. To this date, we have not experienced any adverse events.

### Data analysis

We will perform linear mixed model analyses for repeated measures with between and within subjects to analyse the phenotype such as BP and pulse wave velocity using SPSS (version 25), where fixed effects include time (baseline, after first arm, before second arm, after second arm) and treatment group (placebo versus HAMSAB) [29, 30]. If data is missing, we will use multiple imputation in SPSS. Gut microbiome will be analysed using QIIME2 [26] and other in-house bioinformatics pipelines (such as [19]).

## Discussion

Research into the gut microbiome is evolving at a rapid pace, and in recent years, causal links between the microbiota and disease states, including hypertension, have been discovered. Dietary manipulation via prebiotics increases microbial diversity and SCFA production, which can infer benefits on the host. Translation of findings from pre-clinical models into meaningful results in human studies is important and has been achieved in patients with type 2 diabetes [31]. In regard to hypertension, novel dietary strategies, such as the one described in this trial, are essential, as the impact of cardiovascular disease on the individual and public health systems are immense. The findings from this study will provide the first evidence for the use of a combination of pre- and postbiotics to lower BP in humans.

## Data Availability

Once the study is finished and the data is published, the datasets used during the current study will be available from the corresponding author on reasonable request.

## Abbreviations

ABPM: Ambulatory blood pressure monitoring
BP: Blood pressure
CVD: Cardiovascular disease
HAMS: High amylose maize starches
HAMSAB: Acetylated and butyrylated high amylose maize starch
SCFA: Short-chain fatty acids

## References

[1] Beaney T, Schutte AE, Tomaszewski M, Ariti C, Burrell LM, Castillo RR, Charchar FJ, Damasceno A, Kruger R, Lackland DT, Nilsson PM, Prabhakaran D, Ramirez AJ, Schlaich MP, Wang J, Weber MA, Poulter NR, Napiza-Granada C, Sevilla MRC, Atilano AA, Ona DID, More A, Jose AP, Maheshwari A, Kondal D, Yu W, Li W, Xu S, Yu J, Zhang H, Widyantoro B, Turana Y, Situmorang TD, Sofiatin Y, Barack R, Lin HJ, Wang TD, Chen WJ, Sirenko Y, Evstigneeva O, Negresku E, Yousif ME, Medani SA, Beheiry HM, Ali IA, Zilberman JM, Marin MJ, Rodriguez PD, Garcia-Vasquez F, Kramoh KE, Ekoua D, Lopez-Jaramillo P, Otero J, Sanchez G, Narvaez C, Accini JL, Hernandez-Hernandez R, Octavio JA, Morr I, Lopez-Rivera J, Ojji D, Arije A, Babatunte A, Wahab KW, Fernandes M, Pereira SV, Valentim M, Dzudie A, Kingue S, Djomou Ngongang DA, Ogola EN, Barasa FA, Gitura B, Malik FTN, Choudhury SR, al Mamun MA, Minh VH, Viet NL, Cao Truong S, Ferri C, Parati G, Torlasco C, Borghi C, Goma FM, Syatalimi C, Zelveian PH, Barbosa E, Sebba Barroso W, Penaherrera E, Jarrin E, Yusufali A, Bazargani N, Tsinamdzgvrishvili B, Trapaidze D, Neupane D, Mishra SR, Jozwiak J, Malyszko J, Konradi A, Chazova I, Ishaq M, Memon F, Heagerty AM, Keitley J, Brady AJB, Cockcroft JR, McDonnell B, Lanas F, Chia YC, Ndhlovu H, Kiss I, Ruilope LM, Ellenga Mbolla BF, Milhailidou AS, Woodiwiss AJ, Perl S, Dolan E, Azevedo V, Garre L, Boggia JG, Lee VWY, Kowlessur S, Miglinas M, Sukackiene D, Wainford RD, Habonimana D, Masupe T, Ortellado J, Wuerzner G, Alcocer L, Burazeri G, Sanchez Delgado E, Lovic D, Mondo CK, Mostafa A, Nadar SK, Valdez Tiburcio O, Leiba A, Dorobantu M, de Backer T, Chifamba J, Stergiou G, Nwokocha CR, Sokolovic S, Toure AI, Connell KL, Khan NA, Burger D, de Carvalho Rodrigues M, Kramer BK, Schmieder RE, Unger T, Wyss FS, Yameogo NV, Beistline H, Kenerson JG, Alfonso B, Olsen MH, Soares M (2018). May Measurement Month 2017: an analysis of blood pressure screening results worldwide. Lancet Glob Health.

[2] Vrijens B, Antoniou S, Burnier M, de la Sierra A, Volpe M (2017). Current situation of medication adherence in hypertension. Front Pharmacol.

[3] Burnier M, Egan BM (2019). Adherence in hypertension. Circ Res.

[4] Marques FZ, Mackay CR, Kaye DM (2018). Beyond gut feelings: how the gut microbiota regulates blood pressure. Nat Rev Cardiol.

[5] Reynolds A, Mann J, Cummings J, Winter N, Mete E, Te Morenga L (2019). Carbohydrate quality and human health: a series of systematic reviews and meta-analyses. Lancet.

[6] Whelton SP, Hyre AD, Pedersen B, Yi Y, Whelton PK, He J (2005). Effect of dietary fiber intake on blood pressure: a meta-analysis of randomized, controlled clinical trials. J Hypertens.

[7] Wang X, Ouyang Y, Liu J, Zhu M, Zhao G, Bao W (2014). Fruit and vegetable consumption and mortality from all causes, cardiovascular disease, and cancer: systematic review and dose-response meta-analysis of prospective cohort studies. BMJ.

[8] Marques FZ, Nelson E, Chu PY, Horlock D, Fiedler A, Ziemann M, Tan JK, Kuruppu S, Rajapakse NW, el-Osta A, Mackay CR, Kaye DM (2017). High-fiber diet and acetate supplementation change the gut microbiota and prevent the development of hypertension and heart failure in hypertensive mice. Circulation.

[9] Murphy MM, Douglass JS, Birkett A (2008). Resistant starch intakes in the United States. J Am Diet Assoc.

[10] Roberts J, Jones GP, Rutishauser IHE, Birkett A, Gibbons C (2004). Resistant starch in the Australian diet. Nutr Diet.

[11] Le Leu RK, Hu Y, Brown IL, Young GP (2009). Effect of high amylose maize starches on colonic fermentation and apoptotic response to DNA-damage in the colon of rats. Nutr Metab.

[12] Thorburn AN, McKenzie CI, Shen S, Stanley D, Macia L, Mason LJ (2015). Evidence that asthma is a developmental origin disease influenced by maternal diet and bacterial metabolites. Nat Commun.

[13] Maslowski KM, Vieira AT, Ng A, Kranich J, Sierro F, Yu D (2009). Regulation of inflammatory responses by gut microbiota and chemoattractant receptor GPR43. Nature.

[14] Macia L, Tan J, Vieira AT, Leach K, Stanley D, Luong S, Maruya M, Ian McKenzie C, Hijikata A, Wong C, Binge L, Thorburn AN, Chevalier N, Ang C, Marino E, Robert R, Offermanns S, Teixeira MM, Moore RJ, Flavell RA, Fagarasan S, Mackay CR (2015). Metabolite-sensing receptors GPR43 and GPR109A facilitate dietary fibre-induced gut homeostasis through regulation of the inflammasome. Nat Commun.

[15] Daugirdas JT, Nawab ZM, Jainwttao S, Klok M (1987). Acetate relaxation of isolated vascular smooth muscle. Kidney Int.

[16] Nutting CW, Islam S, Daugirdas JT (1991). Vasorelaxant effects of short chain fatty acid salts in rat caudal artery. Am J Physiol Heart Circ Physiol.

[17] Bartolomaeus H, Balogh A, Yakoub M, Homann S, Marko L, Hoges S (2019). The short-chain fatty acid propionate protects from hypertensive cardiovascular damage. Circulation.

[18] Kim S, Goel R, Kumar A, Qi Y, Lobaton G, Hosaka K, Mohammed M, Handberg EM, Richards EM, Pepine CJ, Raizada MK (2018). Imbalance of gut microbiome and intestinal epithelial barrier dysfunction in patients with high blood pressure. Clin Sci (Lond).

[19] Kaye DM, Shihata W, Jama HA, Tsyganov K, Ziemann M, Kiriazis H (2020). Deficiency of prebiotic fibre and insufficient signalling through gut metabolite sensing receptors leads to cardiovascular disease. Circulation.

[20] Wan Y, Yuan J, Li J, Li H, Yin K, Wang F, Li D (2020). Overweight and underweight status are linked to specific gut microbiota and intestinal tricarboxylic acid cycle intermediates. Clin Nutr.

[21] Maier TV, Lucio M, Lee LH, VerBerkmoes NC, Brislawn CJ, Bernhardt J, et al. Impact of dietary resistant starch on the human gut microbiome, metaproteome, and metabolome. mBio. 2017;8(5):e01343-17. 10.1128/mBio.01343-17 PMC5646248 29042495

[22] David LA, Maurice CF, Carmody RN, Gootenberg DB, Button JE, Wolfe BE, Ling AV, Devlin AS, Varma Y, Fischbach MA, Biddinger SB, Dutton RJ, Turnbaugh PJ (2014). Diet rapidly and reproducibly alters the human gut microbiome. Nature.

[23] Landon S, Colyer C, Salman H. The resistant starch report. Retrieved from Food Australia Supplement. Australia: Goodman Fielder Ltd and National; 2012.

[24] Gill PA, van Zelm MC, Ffrench RA, Muir JG, Gibson PR. Successful elevation of circulating acetate and propionate by dietary modulation does not alter T-regulatory cell or cytokine profiles in healthy humans: a pilot study. Eur J Nutr. 2020;59(6):2651–61. 10.1007/s00394-019-02113-2 31650328

[25] Caporaso JG, Lauber CL, Walters WA, Berg-Lyons D, Huntley J, Fierer N, Owens SM, Betley J, Fraser L, Bauer M, Gormley N, Gilbert JA, Smith G, Knight R (2012). Ultra-high-throughput microbial community analysis on the Illumina HiSeq and MiSeq platforms. ISME J.

[26] Bolyen E, Rideout JR, Dillon MR, Bokulich NA, Abnet CC, Al-Ghalith GA (2019). Reproducible, interactive, scalable and extensible microbiome data science using QIIME 2. Nat Biotechnol.

[27] Marques FZ, Jama HA, Tsyganov K, Gill PA, Rhys-Jones D, Muralitharan RR, Muir J, Holmes A, Mackay CR (2019). Guidelines for transparency on gut microbiome studies in essential and experimental hypertension. Hypertension.

[28] Chan AW, Tetzlaff JM, Gotzsche PC, Altman DG, Mann H, Berlin JA, Dickersin K, Hrobjartsson A, Schulz KF, Parulekar WR, Krleza-Jeric K, Laupacis A, Moher D (2013). SPIRIT 2013 explanation and elaboration: guidance for protocols of clinical trials. BMJ.

[29] Hodgson JM, Croft KD, Woodman RJ, Puddey IB, Fuchs D, Draijer R, Lukoshkova E, Head GA (2013). Black tea lowers the rate of blood pressure variation: a randomized controlled trial. Am J Clin Nutr.

[30] Wellek S, Blettner M (2012). On the proper use of the crossover design in clinical trials: part 18 of a series on evaluation of scientific publications. Dtsch Arztebl Int.

[31] Zhao L, Zhang F, Ding X, Wu G, Lam YY, Wang X, Fu H, Xue X, Lu C, Ma J, Yu L, Xu C, Ren Z, Xu Y, Xu S, Shen H, Zhu X, Shi Y, Shen Q, Dong W, Liu R, Ling Y, Zeng Y, Wang X, Zhang Q, Wang J, Wang L, Wu Y, Zeng B, Wei H, Zhang M, Peng Y, Zhang C (2018). Gut bacteria selectively promoted by dietary fibers alleviate type 2 diabetes. Science.

